# Effects of the Incorporation of Male Honey Bees on Dough Properties and on Wheat Flour Bread’s Quality Characteristics

**DOI:** 10.3390/foods12244411

**Published:** 2023-12-07

**Authors:** Anna Marinopoulou, Georgia Kagioglou, Nikolaos Vacharakis, Stylianos Raphaelides, Maria Papageorgiou

**Affiliations:** Department of Food Science and Technology, International Hellenic University, Alexandrian Campus, 57400 Thessaloniki, Greece; amarinop@food.teithe.gr (A.M.); georgia-ka@hotmail.com (G.K.); nikosvaharakis@gmail.com (N.V.); rafael@food.teithe.gr (S.R.)

**Keywords:** honey bees, insect, drone powder, bread quality, dough rheology

## Abstract

Two different levels (5 and 10%) of male honey bees (drones) in powder form were incorporated into wheat flour, and their impact on dough properties and on bread-quality characteristics were investigated. The incorporation of the drone powder to the wheat flour caused a decrease in the extensibility and energy of the dough in the extensograph and an increase in the dough’s maximum resistance with increasing levels of the added drone powder. The elongational viscosity values of the dough fortified with drone powder were significantly higher than those of the control wheat flour dough. The breads supplemented with 10% drone powder exhibited lower lightness (L*) values compared to the control bread. The addition of drone powder led to an increase in the total dietary fiber content and insoluble dietary fiber content in the fortified bread. Significant differences in the specific volume values were observed between the control bread and the corresponding ones with 10% drone powder. Upon storage, the moisture content of the crumb of the control bread and of the fortified breads were both significantly decreased, while the addition of the drone powder to the wheat flour bread increased the crumb hardness and gumminess but decreased the cohesiveness of the breads.

## 1. Introduction

Over the last few years, there has been a growing interest in enhancing the nutritional value of foods [1] and in reducing their environmental footprint [2]. For this purpose, food industries face the challenge of developing novel food products based on nutrients and bioactive compounds [3]. Legumes and pulses are considered a low-cost source of plant-based proteins, not only because they contain high amounts of protein, but also because they have been cultivated worldwide for years [3]. Thus, legumes and pulses have been used in many bakery products in order to improve their quality and nutritional value [4,5,6]. In this context, many researchers have investigated wheat flour fortification with other plant origin sources [7], such as tomato seed flour and olive pulp [8], and have reported that fortified breads exhibit an acceptable sensory quality and enhance the quality characteristics.

Nowadays, entomophagy is gaining an increasing interest in Western countries [9]. The European Union Novel Food Regulation (EU) 2015/2283 [6,10,11] has recognized insects as novel foods. Edible insects are consumed in Thailand, China, and Japan [12]. The most common edible insects are cockroaches and termites, beetles, flies, cicadas, stink bugs, bees, wasps, ants, butterflies, moths, dragonflies, crickets, and grasshoppers. Many edible insects, and insect-derived products, have been used as an alternative source of protein and other nutritional values [13]. Insects have high contents of proteins (35–61%), fats (15–40%), and minerals (3–10%) [13,14,15], and they are considered a natural source of bioactive compounds with antibacterial, antiviral, and anti-inflammatory properties [16]. Moreover, insects are richer in proteins than beans, lentils, and soybeans, containing 23.5%, 26.7%, and 41.1% protein, respectively [17]. According to Rumpold & Schlüter [13], the main components of insects are proteins and fats, followed by fiber. Edible insects have a significant amount of fiber [14], while the insoluble chitin is considered the most common polysaccharide found in the exoskeleton of most insects. Dietary fibers in edible insects can vary among species. Cricket powder, for instance, consists of 8.5% dietary fibers derived from its chitinous exoskeleton. Among various insects, the African migratory locust is noted for its high fiber content (27%), whereas the Jamaican field cricket is recognized for having a lower content (8%) [18].

Despite the nutritional and therapeutic value of insects, there are also many environmental advantages and sustainable benefits of their utilization [19], i.e., insects can convert proteins into insect proteins much more economically than the livestock species [20]. On the other hand, there are also risks associated with the consumption of edible insects related to allergic reactions or food poisoning due pesticide residues and microbial and heavy metal contamination [21].

Over the last decade, the replacement of wheat flour with edible insect flours such as mealworms, worms, and crickets has been investigated in the literature [22,23,24,25]. Recently, Zielinska et al. [3] studied the physical characteristics and consumer acceptance of bakery products such as muffins containing different levels of cricket flour, while Nissen et al. [26] investigated the incorporation of cricket powder in sourdough gluten-free breads. It has been proven that the substitution of wheat flour with insect flour can affect rheological properties, resulting in the production of bakery products with an improved nutritional value and sensory acceptance [11,27].

It is noteworthy that, to the best of our knowledge, there are no studies in the literature focusing on the effects of incorporating male honey bees (drones, *Apis mellifera*) into wheat bread as an alternative protein source. Honey bee drone brood (DB) (larvae, pupae, and eggs of the honey bee *Apis mellifera*) is considered a promising edible resource and is suggested for human consumption, according to the submission made by the Finnish beekeeper’s association for approval by the European Food Safety Authority [28,29]. Male honey bee (drone) broods contain high amounts of proteins, fatty acids, minerals, and vitamins B3 and B5 [30,31] and can be used as food for human consumption, compared to worker bee broods, due to their limited socio-biological role [32]. In particular, since they do not participate in honey production [33], they are actually killed or evicted by worker broods [34]. Moreover, drones do not bear a poisonous sting such as honey worker bees do [35]; thus, they are not in a position to cause any harm to humans. In this direction, the present study was conducted in order to investigate and evaluate the effects of incorporating drone powder in dough and the effects on the physicochemical and textural properties of wheat bread. In addition, the impact of the replacement of wheat flour with drone powder on the quality of bread during staling was considered worth investigating.

Overall, it is believed that the use of drone powder as a substitute for wheat flour can contribute to the production of innovative functional bakery products with enhanced nutritional and quality characteristics.

## 2. Materials and Methods

### 2.1. Materials

**Figure 1:**
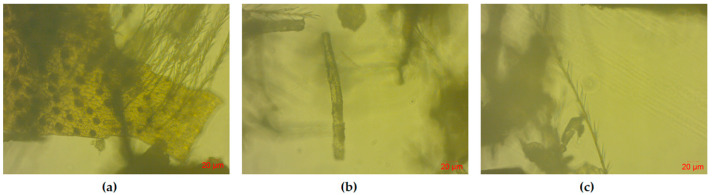
Optical images from the body of the drones ((**a**) abdomen; (**b**) leg; (**c**) hair).

Wheat flour (70% of milling yield), salt, and yeast were purchased from a local market. The drones were collected by a beekeeper in the area of Aridaia (west of Northern Greece). Figure 1 shows optical images from the body of the drones (abdomen, leg, and hair). The morphological structure of the body parts of the *Apia mellifera* drone is described in more detail in studies by Khan & Liu [36] and Gazizova et al. [37]. To prepare the drone in powder form, the insects were frozen (after removing antennae, legs, and wings) and lyophilized in a freeze dryer (Martin Christ). After drying, the drones were ground into powder with a pestle and mortar, packed in polyethylene bags, and stored at ambient temperature. All other reagents and chemicals used were of analytical reagent grade.

### 2.2. Optical Microscopy of the Drones

Microscopic examination of the body parts of *Apis mellifera* drones was conducted using a Zeiss LSM 700 confocal laser microscope (Carl Zeiss, CZ Microscopy GmbH, Jena, Germany) operating in the optical mode with a 10× objective.

### 2.3. Proximate Composition of Raw Material (Wheat Flour and Drone Powder) and Formulated Breads

The moisture content of the wheat flour, drone powder, and formulated breads with different levels of drone powder was gravimetrically determined using the AACC 44-15 standard method [38]. Ash and fat content were analyzed following the ICC Standard 104/1 [39] and AACC Method No. 30-10.01 [40], respectively. Protein content was determined according to the Kjeldahl method [39,41], and it was calculated using 5.7 as a converting factor for wheat flour [8] and 6.25 for drone powder [42]. Total dietary fibers (soluble and insoluble) were determined by the enzymatic method [43], using the KDFR-100A/K-TDFR-200A kit from Megazyme.

### 2.4. Functional Properties of Wheat Flour and Drone Powder

#### Swelling Capacity (SWC)

The swelling capacity (SWC) of the wheat flour and drone powder was measured based on the official method of the AACC Method [44] and of Rana et al. [45], with some modifications. Approximately, 1 g of the sample was placed in a 50 mL graduated cylinder, and 30 mL of distilled water was added. The samples were allowed to stand for 24 h. The swelling capacity was calculated as the ratio of the volume occupied by the sample powder to the initial sample weight.

The swelling capacity was calculated using the following equation:

(1) $$Swelling\;Capacity\left(\frac{mL}{g}\right)=\frac{Volume\;occupied\;by\;sample\;\left(mL\right)}{Initial\;sample\;weight\;\left(g\right)}$$

### 2.5. Rheological Properties of the Dough

#### 2.5.1. Extensiograph Test

The preparation of the control dough (0% of drone powder) was made in a farinograph (Brabender, Duisburg, Germany) by mixing 300 g of wheat flour (14% moisture) with 6 g of NaCl. As was previously mentioned, in the fortified doughs, the wheat flour was replaced with drone powder at 5 and 10% addition levels. Extensiograph tests were conducted using a Brabender extensiograph (Brabender, Duisburg, Germany), following the ICC standard 114/1 [41], after 45, 90, and 135 min of resting time. The parameters obtained from the extensiograph curves were resistance to extension up to 50 mm (R_50_, BU), extensibility (E_x_, mm), and energy (A, cm^2^). The resistance to extension up to 50 mm (R_50_, BU) is defined as the height of the extensiograph curve in BU, after 50 mm of stretching length. The energy (A, cm^2^) is defined as the area under each curve (surface area) in cm^2^, and the extensibility (E_x_, mm) is defined as the length of the extensiograph in mm. In the case of the control dough, the water absorption for the extensiograph was 61.4% and in the fortified dough with 5 or 10% was 60.4% and 61.2%, respectively. The amount of water was determined using the farinograph and is referred to the volume of water required to produce dough with a consistency of 500 farinograph units (BU) in the presence of 2% NaCl, as dictated by the ICC standard 114/1 [41].

#### 2.5.2. Lubricated Squeeze Flow Experiments

Lubricated squeeze flow experiments were carried out following the procedure described by Bousi et al. [46]. The dough samples, as prepared in the extensiograph, were molded using custom-built molds made of aluminum alloy that consisted of two semicircular parts which, when they were attached to each other, formed a ring with an internal diameter of 0.051 m and a height of 0.0098 m. The specimens were transferred on a glass plate with dimensions 0.2 × 0.2 m. To ensure plug-flow conditions during compression of the specimens, the glass plate at the point of location of the sample and the upper surface of the sample were covered with a thin layer of paraffin oil. Then, the two halves of the mold were removed. The samples were uniaxially compressed to 50% of their initial height using a Texture Analyser TA.XT.plus (Stable Micro Systems Ltd., Godalming, UK), equipped with a 30 kg maximum load force cell. The compression tests were performed employing the constant strain rate mode. Four series of measurements were performed at different constant strain rates, i.e., nominal values of 1%, 4%, 7%, and 10%, whereas the compression speed employed was set at 0.01 mm/s. The measurements were carried out at ambient temperature (25 °C).

The calculation of elongational/extensional viscosity was based on the assumption that homogeneous deformation pertained, i.e., perfect slip of the sample at the boundary was achieved.

The biaxial strain rate ${\dot{\varepsilon }}_{b}i$ is defined as [47]

(2) $${\dot{\varepsilon }}_{b}=\frac{1}{2}{\dot{\varepsilon }}_{T}$$

(${\dot{\varepsilon }}_{T}$ is defined as the instantaneous strain rate), when a steady flow is achieved, i.e., stress and strain rate reach constant values. Then, the elongational viscosity $n_{B}\left({\dot{\varepsilon }}_{b}\right)$ was calculated as the ratio of stress to biaxial strain rate, based on the following equation [48]:

(3) $$n_{B}\left({\dot{\varepsilon }}_{b}\right)=\underset{t\to \infty }{\lim}\left[n_{B}^{+}\left(t,{\dot{\varepsilon }}_{b}\right)\right]$$

The mathematics employed to derive Equations (2) and (3) is described in studies by Kontou et al. [49].

### 2.6. Breadmaking

The preparation of the control bread (100% wheat flour) and the fortified breads with drone powder was made based on the following formulations: the control bread’s recipe contained 100% wheat flour (300 g, 14% on a moisture basis), salt (2% onaflour basis), dried yeast (1.5% on a flour basis), and water (184.2 mL). The breads containing drone powder were prepared by replacing the wheat flour with 5 and 10% (*w*/*w*) of drone powder and by adjusting the water, according to Section 2.5.1. A two-step bulk fermentation and proofing were used. Initially, all ingredients were mixed using a mixer (Samix R, Dito-Sama, France) for 3 min at medium speed, followed by 2 min at high speed. Then, the doughs were fermented at 32 °C and ~70% relative humidity for 30 min. After fermentation, the doughs were hand-molded, cut into three equal pieces, placed into tin pans, and proofed for a further 50 min at 32 °C. Then, the doughs were baked in a laboratory oven (Neff, Bruchsal, Germany) at 230 °C for 45 min. All loaves of bread were allowed to cool down at ambient temperature until constant weight, with each loaf weighing approximately 130 g. After cooling, the following measurements were carried out: loaf volume (determined by rapeseed displacement), specific volume (by dividing loaf volume by weight), ash content [39], and crumb color of the breads. Finally, the breads were packed in polyethylene bags and were stored at 4 °C for 1, 4, and 6 days until analysis.

### 2.7. Bread Characterization

#### 2.7.1. Mechanical Properties of the Crumb

A texture profile analysis (TPA) was carried out, according to the AACC Method 74-09 [38], using a texture analyzer TA-XT plus (Stable Micro Systems, Gudaiming, Surrey, UK), equipped with a P/50 probe. A two-bite TPA test was employed in order to measure the texture parameters, i.e., the hardness, cohesiveness, and gumminess of the bread crumbs. Hardness is defined as the peak strength observed during the first compression of the bread crumb. Cohesiveness was determined as the ratio of the positive force area observed during the second compression to that during the first compression. Gumminess was calculated by multiplying the hardness and cohesiveness values. The tests were performed by applying a 40% strain to the sample specimens obtained from the crumb of the slices in the form of cylindrical disks. The thickness of the samples was 2.5 cm and the diameter 3.5 cm. The test speed was set to 2.0 mm/s. The compression tests were carried out after 1, 4, and 6 days of storage at 4 °C. At least three measurements were conducted for each formulation. Measurements were carried out at ambient temperature (25 °C).

#### 2.7.2. Color Analysis of the Crumb Color

The color of bread crumbs was measured using a non-contact imaging spectrophotometer (Meta Vue VS3200, X-Rite, Grand Rapids, MI, USA). Crumb color parameters (L*, a*, b*) and the yellowness index were determined. L* denotes lightness and ranges from 0 (black) to 100 (white), a* shows the redness/greenness value and ranges from −60 (greenness) to +60 (redness), and b* indicates the yellowness/blueness value which ranges from −60 (blueness) to +60 (yellowness). The yellowness index shows the color changes of a sample from clear or white towards yellow. Color analysis measurements were carried out only for the control breads and fortified breads with 10% drone powder in order to investigate, compare, and evaluate the changes of the color of the breads containing zero and the highest addition level of drone powder. All measurements were run in triplicate.

### 2.8. Statistical Analysis

Data were analyzed using Minitab 18 Statistical Software (Minitab Inc., State College, PA, USA) and JMP Statistical Software (JMP 13.2.1., SAS Institute Inc., Cary, NC, USA, 2016). Results are expressed as mean values with standard deviation. Data were submitted to a one-way analysis of variance (ANOVA),and a Tukey’s pairwise comparison of means was performed in order to investigate whether means are significantly different (*p* < 0.05). Differences between the mean values of chemical components were evaluated by the Paired Samples Test (2-tailed). Interval plots were used to describe the 95% confidence intervals based on the pooled error of ANOVA. A Principal Component Analysis (PCA) [50] and a cluster analysis (CA) were conducted in order to detect potential relationships among variables as affected by storage time.

## 3. Results

### 3.1. Proximate Chemical Composition and Functional Properties of Wheat Flour and Drone Powder

The chemical composition and the functional properties (swelling capacity) of the wheat flour and drone powder are shown in Table 1. The wheat flour exhibited higher moisture content values than the drone powder. On the other hand, the drone powder had significantly higher fat (over 4 times), ash (over 1.5 times), and protein (over 6 times) content than the wheat flour. It is well known that edible insects consist mainly of proteins and fats, followed by fiber, non-fiber carbohydrates, and ash [13]. According to Hrassnigg&Crailsheim [51], the protein content of drones in various development stages ranges from 40.3 to 79.4%. High protein, fat, and ash content values were also reported by Ghosh et al. [42] in the chemical composition of worker, larvae, pupae, and adults of *Apis mellifera ligustica*. Moreover, as shown in Table 1, the drone powder presented higher soluble and insoluble dietary fiber values compared to the wheat flour. However, no statistical difference was observed between the soluble dietary fiber content in the wheat flour and drone powder. Regarding the swelling capacity of the raw materials, it should be noted that the swelling capacity of the drone powder was significantly higher than of the wheat flour. These results are in agreement with those of Aguilera et al. [52], who studied the chemical composition of six species of insects and reported that the insect flours were characterized by high values of the swelling capacity, which is related to the high protein content of insect flours.

### 3.2. Extensional Properties of the Doughs

Table 2 shows the extensional properties of the control dough (0%, drone powder) and of the doughs incorporated with 5 or 10% drone powder. The results reveal that the replacement of wheat flour with drone powder affected the rheological behavior of the supplemented doughs. The addition of drone powder resulted in an increase of the resistance to extension up to 50 mm (R_50_) and a decrease of extensibility (E_x_) for all testing periods. The elongational energy (A) also decreased with an increase in drone powder concentration. These differences are probably related to the presence of high amounts of dietary fibers and proteins of the drone powder which might dilute the gluten network [23,27,53]. This is more pronounced in the enriched wheat flour with the drone powder at 10% concentration, indicating a greater weakening of the gluten network. In addition, the increase in dietary fibers might also affect the dough extensibility [54]. The decrease in the R_50_ value, which was also observed after 135 min of resting time, could be also attributed to the weakening of the gluten network. A decrease in the dough extensibility was also reported by Khatun et al. [55], who investigated the biaxial extensibility of wheat flour dough enriched with the flour or paste of house crickets (*Acheta domesticus*).

### 3.3. Elongational Viscosity Determination

**Figure 2:**
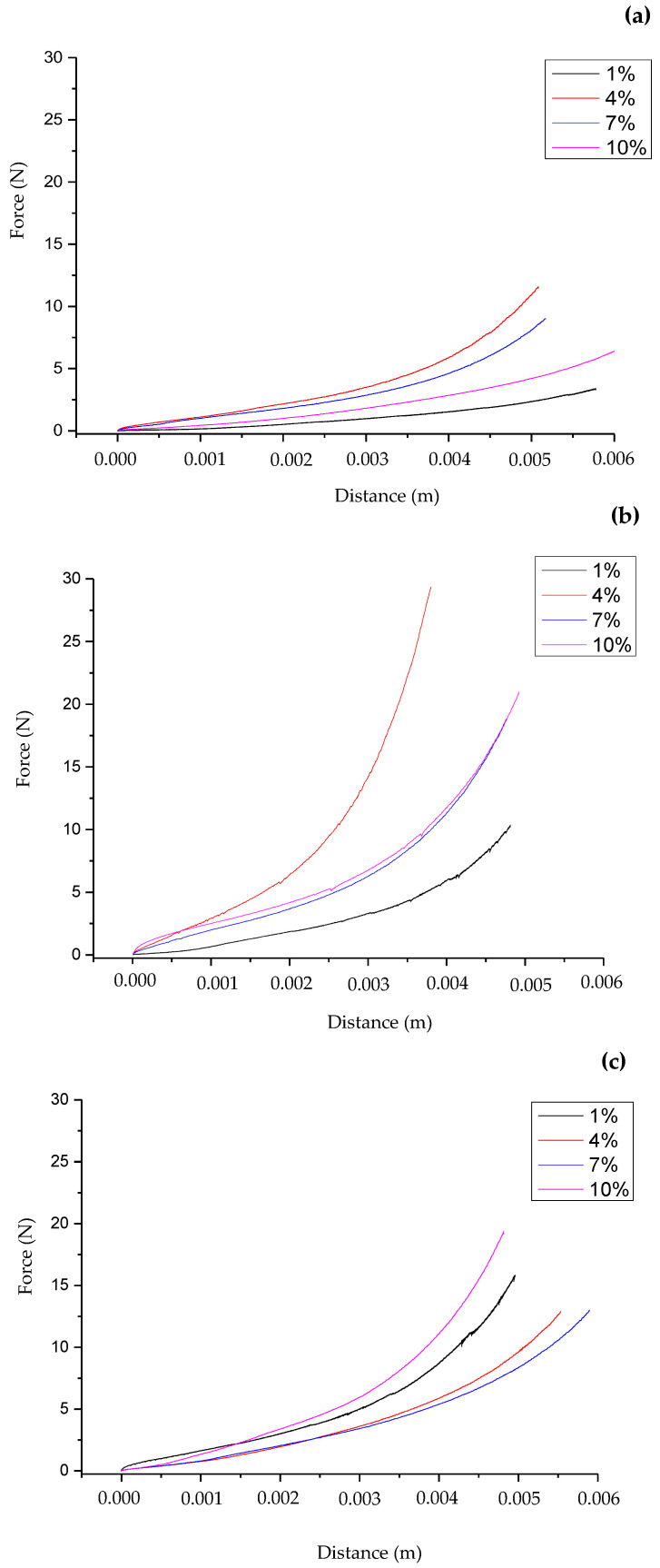
Force–distance curves of (**a**) control dough (0% of drone powder) and of doughs containing (**b**) 5% and (**c**) 10% drone powder, under constant strain rate (nominal) values 1.0, 4.0, 7.0, 10.0%.

**Figure 3:**
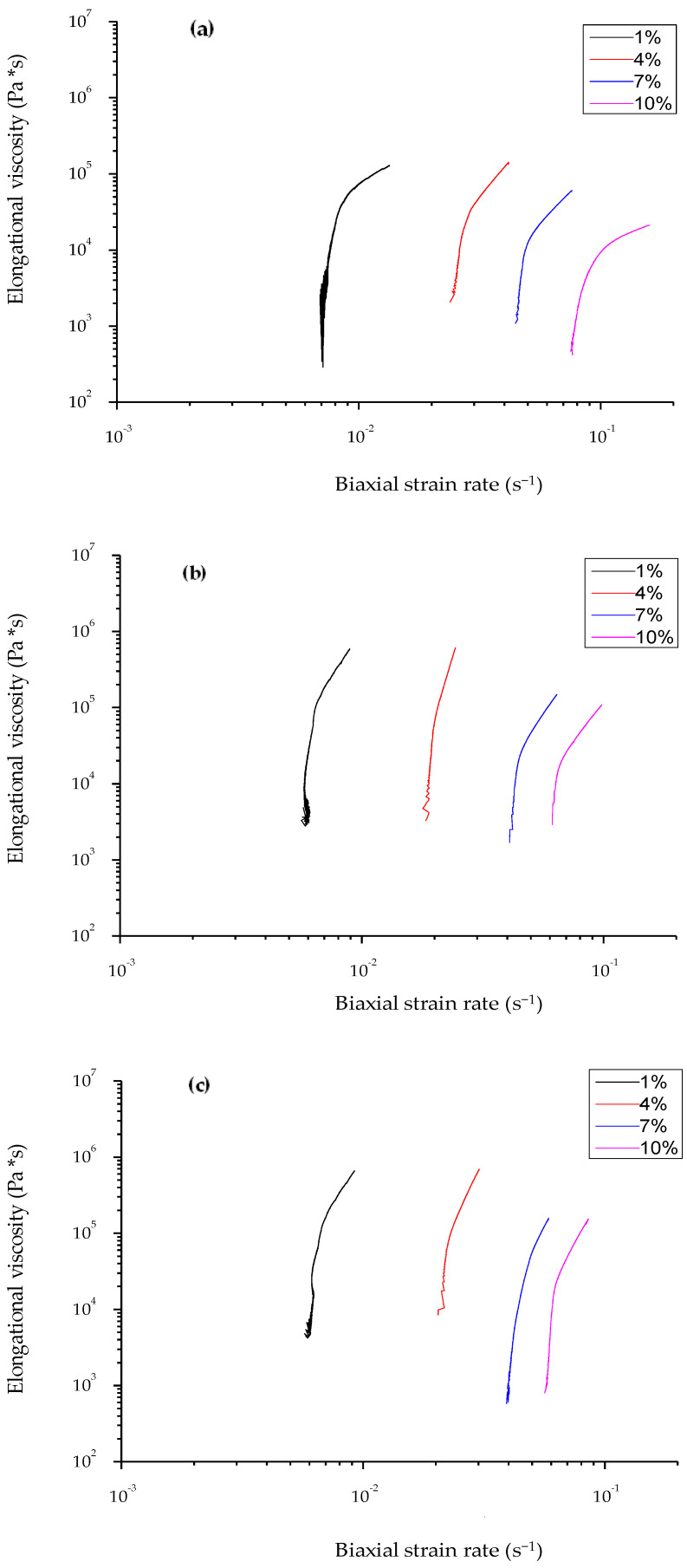
Elongational viscosity flow curves as a function of biaxial strain rate (nominal) values 1, 4, 7, and 10% of (**a**) control dough (0% of drone powder)and of doughs containing (**b**) 5% and (**c**) 10% drone powder.

**Figure 4:**
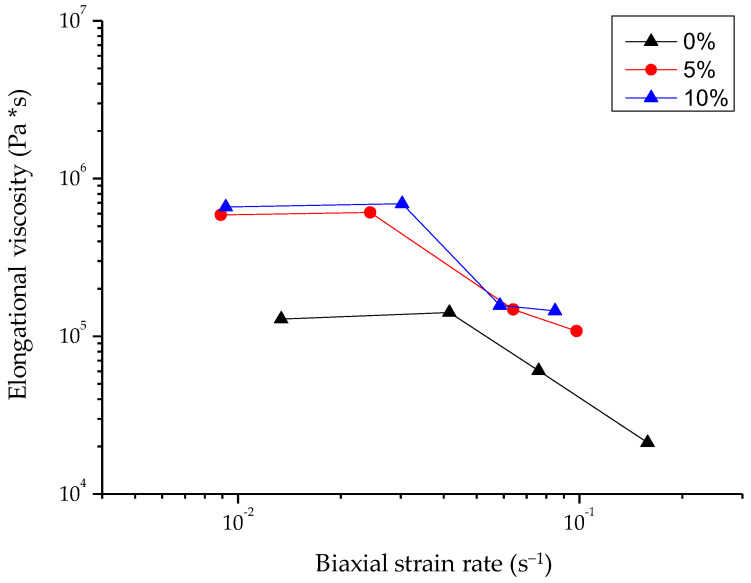
Elongational viscosity as a function of biaxial strain rate of control dough (0% of drone powder) and of doughs containing 5% and 10% drone powder under controlled strain rate.

Figure 2 shows typical force–distance curves of the wheat flour dough and of the enriched dough with 5 or 10% drone powder at four different strain rate values, nominally designated as 1, 4, 7, and 10%. The elongational viscosity–biaxial strain rate curves for all doughs are displayed in Figure 3, while the elongational viscosity curves as a function to the corresponding values of the biaxial strain rate are shown in Figure 4. It should be noted that the elongational viscosity values, as presented in Figure 4, were calculated from the plateau section of the flow curves, which is referred to as the maximum values of the elongational viscosity. As shown in Figure 2, higher force values were recorded for the doughs containing drone powder than the control dough. All doughs exhibited Newtonian behavior at low biaxial strain values and pseudoplastic behavior at higher values. In all doughs, the elongational viscosity was decreased with the increase of the biaxial strain rate (Figure 3). According to Figure 4, the wheat flour replacement with drone powder resulted in an increase in elongational viscosity. This could be attributed to the water-binding capacity of the dough which, in turn, increased the degree of bonding in the protein network. This was also verified by the results obtained from the swelling capacity values of the drone powder (Section 3.1., Table 1), which show that the drone powders had significantly higher swelling capacities than the wheat flour. It should be noted that the fortified dough with 5 and 10% drone powder exhibited similar elongational viscosity values, which indicates the lack of more available water molecules to bind with the proteins. Based on the above, it is inferred that the elongational viscosity was most likely affected by the protein network rather than the different addition levels of drone powder.

### 3.4. Physicochemical Characteristics of Bread

#### 3.4.1. Specific Volume, Ash Content, and Dietary Fibers and Color Analysis of Bread Crumb

**Figure 5:**
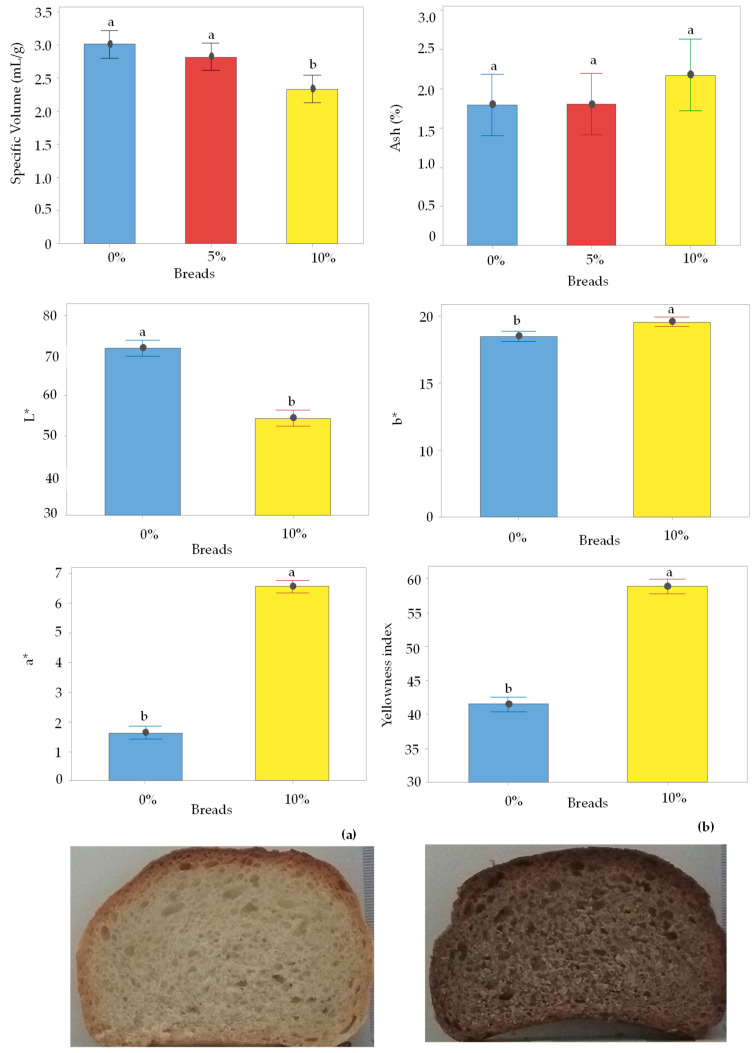
Specific volume (mL/g) and ash (%) content of control bread (0% of drone powder) and of breads containing drone powder at different levels (5 or 10%). Color parameters, L*, a*, b*, and yellowness index, of control bread (0% of drone powder) and of breads containing 10% drone powder. Images of (**a**) control bread (0% of drone powder) and of (**b**) breads containing 10% drone powder. Vertical lines denote the 95% confidence interval of means based on the error mean square of ANOVA. Groups that do not share a letter have a mean difference that is statistically significant (*p* < 0.05).

Figure 5 shows the specific volume, ash content, and color parameters of the control bread and of the breads containing drone powder. Specific volume values ranged from 3.01 to 2.34 mL/g. Statistical differences were recorded in the specific volume values, indicating that the partial substitution of wheat flour with the drone powder probably affected the gas retention properties of the gluten network. High specific volume values indicate the formation of a more open-cell crumb structure with more gap cells andasofter texture [56]. Bartkiene et al. [57] reported that the addition of 5% cricket flour did not significantly affect the specific volume of bread, while the addition of higher levels of insect powder resulted in a decrease of the specific volume. Cappelli et al. [11] and Khuenpet et al. [58] found that high levels of cricket or mealworm powder negatively affected the specific volume of wheat bread. On the other hand, Kowalski et al. [22] observed that the addition of mealworm powder flour improved the volume of wheat bread. It should be noted that the observed differences in the specific volume of the enriched bread with mealworm powder flour may be related to the different proportions of insect flour used for the preparation of the breads. Thus, it can be inferred that the specific volume of the breads can be affected by the different insect flour-inclusion levels or the type of edible insect flour, and the replacement of the wheat flour with insect flour could result either in an increase or a decrease in the specific volume of bread. Similar ash content values were found between the control bread and the breads enriched with drone powder (Figure 5). Mafu et al. [25] reported that the ash content of bread enriched with cricket powder increased with an increase in the insect powder level, while no differences were recorded in the ash content in muffins enriched with cricket powder [24]. The content of total soluble dietary fiber (TDF), insoluble dietary fiber (IDF), and soluble dietary fiber (SDF) are shown in Table 3. The results reveal that the wheat flour substitution with drone powder resulted in an increase indietary fiber, that is in the breads containing 5% drone powder, the total dietary fiber increased by 12.41%, while in the breads with 10% drone powder, the total dietary fiber increased by 27%. The increase in the TDF for the fortified breads compared to the control bread could be assigned to the high content of dietary fibers of the drone powder (Table 3). As mentioned above, insects are rich in dietary fibers, mainly insoluble [59]. These results are in agreement with those of González et al. [23], who found that the TDF in breads enriched with insect flours were higher than a control bread. Therefore, it can be said that the addition of drone powder probably contributed to an increase in the total soluble dietary fiber, implying that insect flours could also be used as an alternative source of dietary fibers. Comparing the dietary fiber content of the drone-enriched breads (Table 3) to that of the drone powder (Table 1), it can be inferred that there is a decrease in the amount of IDF and an increase in the SDF. This was expected since many thermal processes such as boiling, pressure cooking, roasting, frying, etc., can partially convert the insoluble dietary fiber into a soluble one [60]. A similar observation was also made by Koletta et al. [61] in the case of wheat breads enriched with wholegrain non wheat flours. The authors reported that the baking process led to an increase in the soluble dietary fibers, which was probably due to the partial conversion of insoluble dietary fibers into soluble dietary fibers.

Images of the control bread (0% of drone powder) and of the breads containing 10% drone powder and the color parameter values (L*, a*, b*, and yellowness index) of the breads are presented in Figure 5. The breads containing the drone powder exhibited lower L* values compared to the control breads. Similar results in breads fortified with insect flours were also reported by many researchers [3,23,27]. The breads containing the drone powder showed significantly higher a* and b* values than the control bread, indicating that the drone-enriched breads were more reddish and yellowish in color than the control. Higher yellowness index values were also recorded for the breads fortified with drone powder. Considering that the conditions applied during baking were similar for both products, it could be said that these differences are related to the addition of the drone powder to the wheat flour. Most likely, the darkening of the crumb could be related to the high protein content, resulting in an increase in the Maillard browning reaction [25]. These differences could also be related to the color of the raw material used for the preparation of the breads [3]. That is, since the color of the drone powder is brownish black [62], the color of the crumb of the fortified bread is expected to be darker than the control bread. Similarly, González et al. [23] reported that the breads containing Hermetiaillucens displayed a darker color than those with mealworm Tenebrio molitor or cricket Acheta domestica flours.

#### 3.4.2. Crumb Moisture Content on Staling

**Figure 6:**
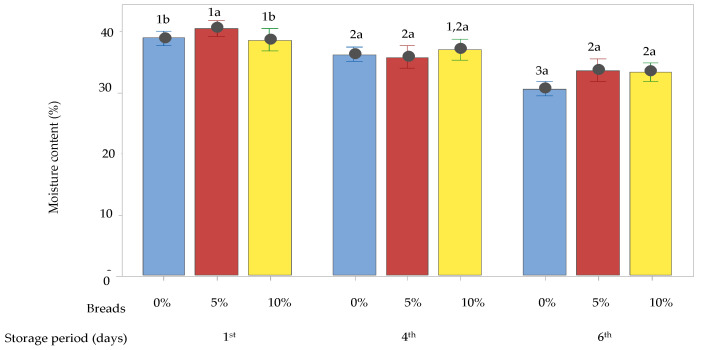
Moisture content (%) of crumb of control bread (0% of drone powder) and of breads containing 5 or 10% drone powder, after 1, 4, and 6 days of storage. Vertical lines denote the 95% confidence interval of means based on the error mean square of the analysis of variance. Different letters within the same day group or numbers for the same recipe (on different days), used as superscripts, indicate differences (*p* < 0.05) amongst the means, as determined by Tukey’s multiple comparison test.

Statistical differences were reported in the moisture bread crumb content in all the examined samples. Upon storage, the moisture content of the control bread and of the breads containing the drone powder at levels 5 and 10% ranged from 38 to 40%. As shown in Figure 6, the drone-enriched breads had higher moisture content values than the control bread during storage. In detail, the fortified breads with 5% drone powder presented higher moisture content values after 1 day of storage. A significant decrease in moisture content was recorded for all breads after 4 days of storage, while after 6 days of storage, the moisture content decreased only in the case of the control bread. These differences could be assigned to the increased water retention capacity due to the drone proteins and to the high fat content which prevented the evaporation of moisture during baking [23]. It is probable that, upon storage, the addition of the drone powder contributed not only to the better water distribution in the dough systems but also to the controlled loss of water which, in turn, reduced the retrogradation of starch. The positive effect of cricket flour on delaying the staling process was also reported by Ndiritu et al. [63]. On the other hand, Kowalski et al. [22] found that the addition of edible insect flours to wheat flours led to a decrease in crumb moisture, while González et al. [23] found no significant differences in the moisture content between the breads containing insects’ flours and the control breads, which was probably related to the high fat content which prevented the evaporation of water. Similar results were also reported by Cappelli et al. [11].

#### 3.4.3. Texture Profile of Breads

**Figure 7:**
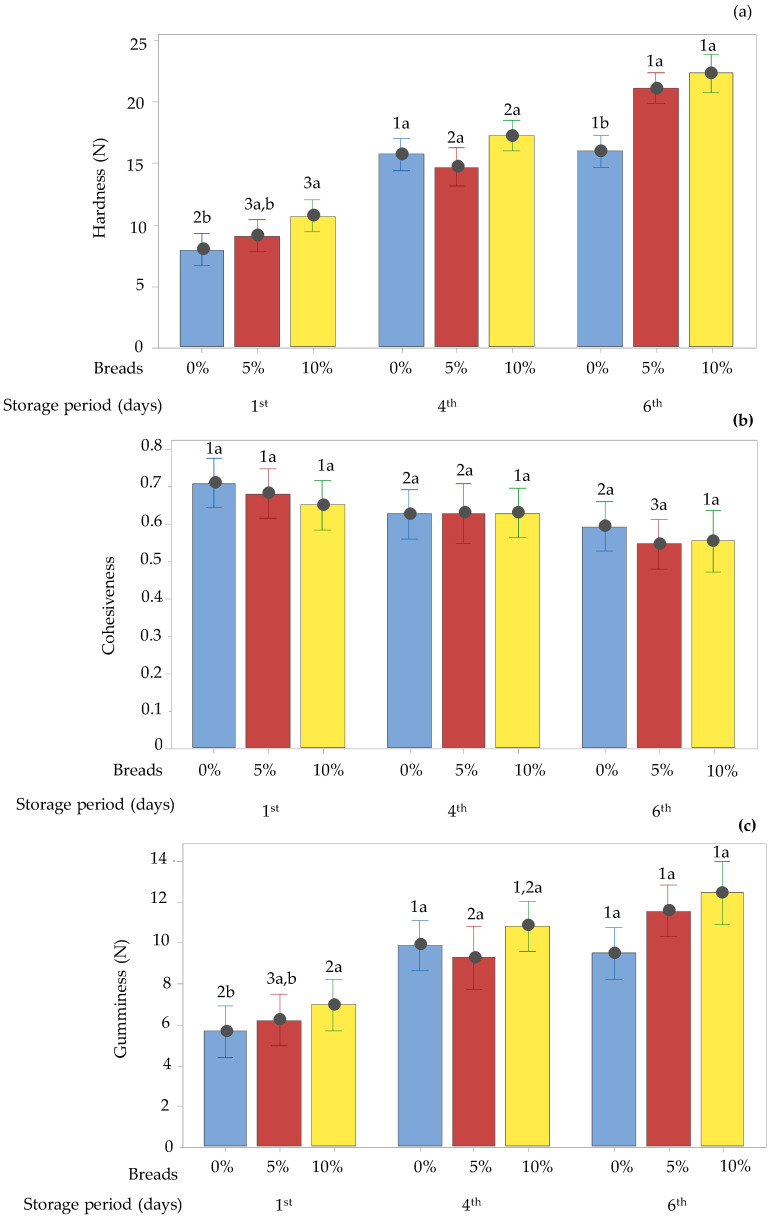
Texture profile parameters: hardness (**a**), cohesiveness (**b**), and gumminess (**c**) of control bread (0% of drone powder) and of breads containing 5 or 10% drone powder, after 1, 4, and 6 days of storage. Vertical lines denote the 95% confidence interval of means based on the error mean square of the analysis of variance. Different letters within the same day group or numbers for the same recipe (on different days), used as superscripts, indicate differences (*p* < 0.05) amongst the means, as determined by Tukey’s multiple comparison test.

The texture parameters (hardness, cohesiveness, and gumminess) of the control breads and of the fortified breads with 5 or 10% drone powder are displayed in Figure 7. The statistical analysis revealed that all the examined parameters were significantly affected by the addition of insect flour. As shown in Figure 7, the fortified breads exhibited higher hardness values than the control bread while having higher moisture content. According to Wahyono et al. [64], the heterogenous cellular structure might affect the textural properties of bread crumbs. It is probable that, during the breadmaking process, the drone powder was not uniformly distributed in the wheat flour dough, resulting in the formation of a heterogenous crumb structure and of protein aggregates [65]. The high hardness values obtained in all samples regardless of their moisture content could be also related to the aggregation of starch chains and the formation of a more crystalline structure, which was probably due to the distribution of water in the various areas of the bread crumb [66]. The incorporation of drone powder into wheat flour can also increase the hardness of the bread crumb due to the delayed hydration of the gluten protein and the development of the gluten network [11]. The increased hardness of the fortified breads could be also related to the higher fiber content which inhibits the dough expansion, resulting in the formation of a more compact structure [67]. Similar to our results, González et al. [23] reported that breads enriched with *H. illucens* had significantly higher moisture and hardness values than wheat flour breads. All breads showed an increase in hardness values (Figure 7) during the storage days. It is well known that bread staling occurs due to moisture loss and the reorganization of amylopectin into a more crystalline structure [66]. Since the moisture content is inversely proportional to firmness [61,68] and the degree of crystallinity of the bread crumb [68], and since the degree of crystallinity is proportional to crumb firmness [66], upon storage, the structure of the crumb becomes more rigid [69] without the presence of air gaps, and, hence, as the moisture content decreases [70], the rigidity and the hardness of the crumb is increased. A similar trend was also recorded in the case of gumminess values of all bread crumbs. As for the cohesiveness, the results showed that there were no significant differences among the breads on the same day of storage. As can be seen in Figure 7, the cohesiveness of the control bread and of the breads containing 5% drone powder decreased during storage. A slight but not statistically significant decrease was noticed in the bread enriched with 10% drone powder. Considering that gluten plays a principal role in breadmaking by improving gas retention and forming a strong network [71], the structure of the gluten network was probably disturbed by the presence of the drone powder and became less continuous. This, in turn, might result in a product with lower cohesiveness [72]. A similar explanation was also reported by other researchers in the case of the addition of plant materials such as quinoa flour or potato pulp as wheat substitutes [73]. Similar to our results, Mafu et al. [25] found that breads enriched with cricket flour were less cohesive than a control bread, and the authors attributed this to the high fat content of the insect flour, which probably affected the texture and gluten network [74]. Moreover, the low cohesiveness values could also be assigned to the weak starch–protein interaction due to the replacement of the gluten protein [75] with the drone protein. Thus, it can be inferred that the replacement of the wheat flour with the drone powder resulted in the production of harder and less cohesive bread than the control bread.

### 3.5. Statistical Analysis

#### 3.5.1. Principal Component Analysis

**Figure 8:**
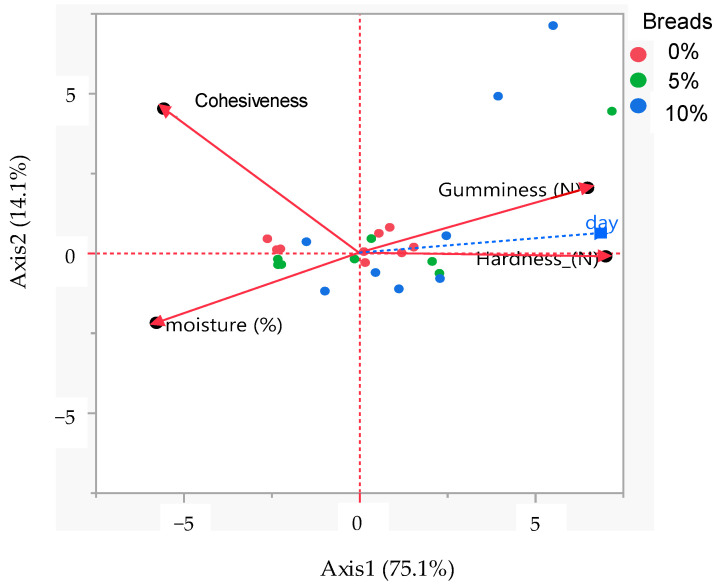
PCA-biplot of the correlation coefficients between the physicochemical and textural attributes of control bread (0% of drone powder) and of fortified breads with 5 or 10% drone powder expressed as the average of the storage days.

The PCA was applied in order to investigate the relationship between the physicochemical (moisture) and texture properties (hardness, gumminess, and cohesiveness) of the breads. The PCA-biplot of the correlation coefficients among the tested attributes is depicted in Figure 8. The correlation coefficients of the aforementioned attributes with two axes are presented in Table 4. In the PCA-biplot, large obtuse angles between variables indicate a negative correlation, while low acute angles indicate a positive correlation. Gumminess and hardness were positively correlated to Axis 1, while cohesiveness and moisture was associated to Axis 2. A strong positive relationship was observed between hardness and gumminess and a negative one between moisture and hardness. On the other hand, gumminess and hardness were closely positioned to the storage days, implying that those attributes are strongly affected during aging. Overall, it should be noted that the PCA results verify the results obtained by the physicochemical and TPA analyses.

#### 3.5.2. Cluster Analysis

**Figure 9:**
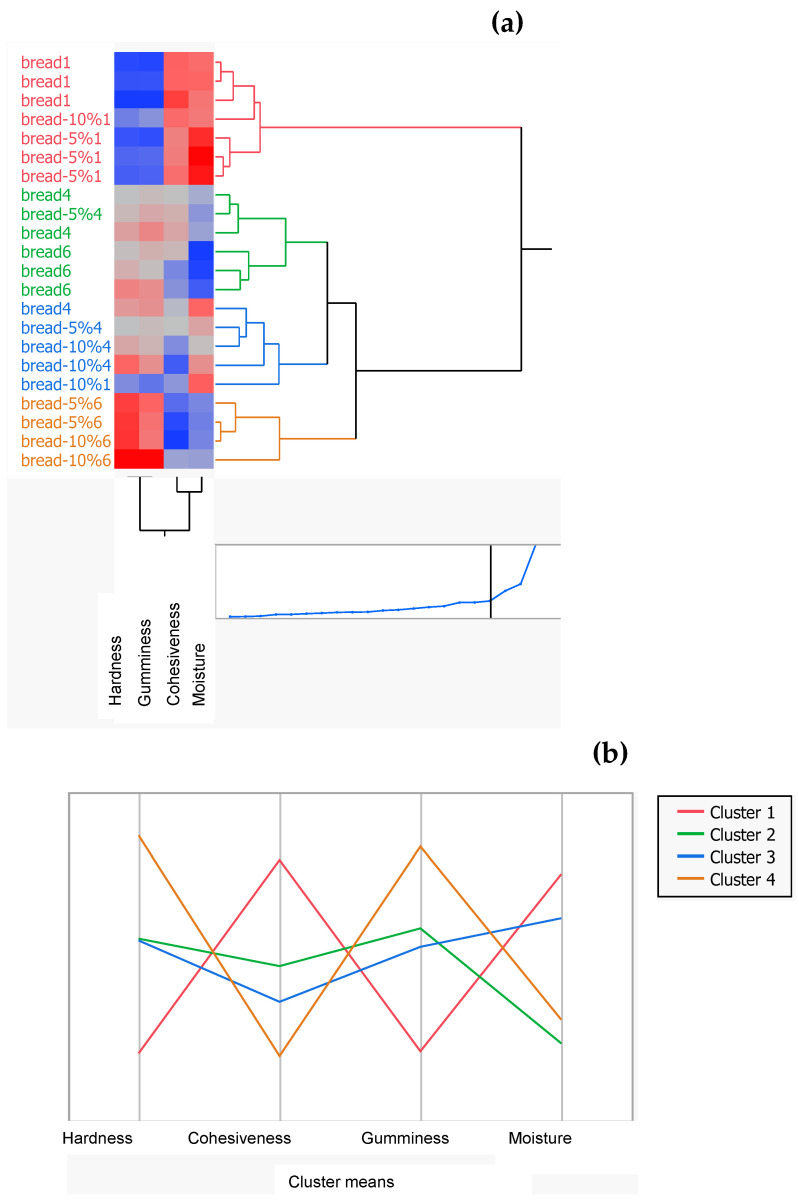
(**a**)Two-way dendrogram of clusters (0% of drone powder) and of breads fortified with 5 or 10% drone powder as a function of storage days; (**b**) array of mean values of attributes within each cluster. In the two-way dendrogram οf clusters, the number 1, 4,and 6 are at the end of the samples.

A two-hierarchical analysis was conducted in order to categorize the physicochemical and mechanical properties of bread during staling. The results obtained from the hierarchical analysis are presented as a dendrogram in Figure 9, while the mean values of the attributes within each cluster are shown in Table 5. In the dendrogram, the blue areas show low intensities of the attributes while the red areas denote high intensities. As shown in Figure 9, the cluster analysis revealed the presence of four clusters (groups). Cluster 1 consisted of the control bread and of the breads containing 5 and 10% drone powder and which were stored for 1 day (colored red). Cluster 2 included the control bread, stored for 4 and 6 days, and of the breads with 5% drone powder, which were stored for 4 days (colored green). On the other hand, cluster 3 comprised the breads enriched with 5 and 10% drone powder after 1 and 4 days of storage and the control breads after 1 day of storage (colored blue). Cluster 4 contained the breads with 5 and 10% drone powder, which were stored for 6 days (colored orange). Cluster 1 was the largest while cluster 4 was the smallest. The breads belonging to cluster 4 exhibited high values of hardness and gumminess. This was expected since cluster 4 contained breads stored for 6 days. In addition, one sample of the breads fortified with 10% drone powder and stored for 1 day was grouped with the breads stored for 4 days in cluster 3, implying that there are close similarities among these samples. Clusters 1 and 3 were characterized by the highest values of hardness. Based on the above results, it is inferred that the physicochemical and textural properties of the breads are strongly affected by the storage days and that there is a strong positive relationship between these attributes.

Overall, it should be noted that the results obtained from the cluster analysis are consistent with those obtained from the PCA.

## 4. Conclusions

Breads fortified with 5 and 10% drone powder at different levels were prepared, and the effects of the incorporation of drone powder on the physicochemical and textural properties of wheat flour bread were evaluated. A chemical analysis revealed that the drone powder exhibited higher fat content, ash content, protein content, swelling capacity, insoluble and soluble dietary fiber content values than the wheat flour. The replacement of the wheat flour with the drone powder resulted in an increase inR_50_, a decrease in A and E_x_, and an increase in the elongational viscosity values, implying that the main component that can affect the rheological properties of dough is drone protein. A progressive increase, albeit not significant statistically, was observed in the ash content of the breads by increasing the drone powder by up to 20%. The breads fortified with 10% drone powder displayed the lowest specific volume. The progressive incorporation of the drone powder resulted in a higher total dietary fiber content and insoluble dietary fiber content in the final breads, thus enhancing their nutritive value. The crumb color of the breads fortified with 10% drone powder was darker than the control bread, which is attributed to the high protein content. Upon storage, the supplemented breads exhibited higher moisture content values than the control breads, while no significant differences were recorded on the same day of storage. Regarding their textural characteristics, the incorporation of the drone powder to wheat flour resulted in the production of harder and less cohesive breads than the control bread. Further research is in progress in order to investigate and explore, in depth, the effects of incorporating higher levels of drone powder on the bread-quality characteristics.

## Figures and Tables

**Table 1:** Chemical characteristics and swelling capacity of wheat flour and of drone powder.

Parameters	Wheat Flour	Drone Powder
Moisture	10.21 ^a^ (±0.61)	8.40 ^b^ (±0.87)
Fat	1.07 ^a^ (±0.11)	5.71 ^b^ (±0.30)
Protein	11.83 ^b^ (±0.27)	30.97 ^a^ (±0.54)
Ash	0.63 ^b^ (±0.03)	4.70 ^a^ (±0.37)
Insoluble dietary fiber (%)	1.82 ^b^ (±0.17)	14.98 ^a^ (±0.17)
Soluble dietary fiber (%)	0.41 ^a^ (±0.05)	0.38 ^a^ (±0.01)
Total dietary fiber (%) ^1^	2.23	15.36
Swelling (mL/g)	2.23 ^b^ (±0.35)	4.85 ^a^ (±0.92)

^1^ Total dietary fiber (%) was calculated as the average of the insoluble and soluble dietary fibers. Different letters in the same line, used as superscripts, indicate differences (*p* < 0.05) amongst the means, as determined by the Paired Samples Test (2-tailed).

**Table 2:** Extensional properties (R_50_, resistance to extension up to 50 mm; A, energy; E_x_, extensibility) of control dough (0%, drone powder) and doughs incorporated with 5% or 10%.

Resting Time	45 min			90 min			135 min		
Samples	R_50_ (BU)	A (cm^2^)	E_x_ (mm)	R_50_ (BU)	A (cm^2^)	E_x_ (mm)	R_50_ (BU)	A (cm^2^)	E_x_ (mm)
Control dough	424	158	192	580	153	172	602	147	155
Dough enriched with 5% drone powder	438	139	169	640	142	139	612	132	140
Dough enriched with 10% drone powder	670	147	138	931	144	109	838	121	104

**Table 3:** Insoluble dietary fiber (IDF), soluble dietary fiber (SDF), and total dietary fiber (TDF) of control bread (0% of drone powder) and of breads containing 5 or 10% drone powder.

Breads	IDF ^1^ (%)	SDF ^2^ (%)	TDF ^3^ (%)
0%	3.26 ^c^ (±0.05)	0.93 ^a^ (±0.03)	4.19
5%	3.86 ^b^ (±0.07)	0.86 ^a^ (±0.03)	4.71
10%	4.44 ^a^ (±0.14)	0.88 ^a^ (±0.04)	5.32

^1^ IDF: Insoluble Dietary Fiber, ^2^ SDF: Soluble Dietary Fiber, ^3^ TDF: Total Dietary Fiber. Data are presented as means ± standard deviation. Means in the same column with different letters were significantly different at *p* < 0.05, as determined by Tukey’s multiple comparison test.

**Table 4:** Correlation coefficients of physicochemical and textural properties with the two major components.

Attributes	Axis1	Axis2
Hardness	0.975	−0.016
Gumminess	0.904	0.282
Cohesiveness	−0.771	0.626
Moisture	−0.800	−0.304

**Table 5:** Mean values of the physicochemical and textural attributes within each cluster.

Cluster	Count	Hardness	Cohesiveness	Gumminess
1	7	8.87	0.696	6.165
2	6	15.63	0.615	9.58
3	5	15.52	0.588	9.07
4	4	21.71	0.545	11.86

## Data Availability

The data used to support the findings of this study can be made available by the corresponding author upon request.
